# An Adaptable Protocol to Generate a Murine Enteroid–Macrophage Co-Culture System

**DOI:** 10.3390/ijms25147944

**Published:** 2024-07-20

**Authors:** Viktoria Hentschel, Deepalakshmi Govindarajan, Thomas Seufferlein, Milena Armacki

**Affiliations:** Department of Internal Medicine 1, University Hospital Ulm, Albert-Einstein-Allee 23, 89081 Ulm, Germany; deepalakshmi.govindarajan@uniklinik-ulm.de (D.G.); thomas.seufferlein@uniklinik-ulm.de (T.S.); milena.armacki@googlemail.com (M.A.)

**Keywords:** co-culture, intestinal organoids, macrophages

## Abstract

Impairment of the intestinal epithelial barrier is frequently seen as collateral damage in various local and systemic inflammatory conditions. The inflammatory process is characterized by reciprocal interactions between the host intestinal epithelium and mucosal innate immune cells, e.g., macrophages. This article provides step-by-step instructions on how to set up a murine enteroid–macrophage co-culture by culturing cellular elements in proximity separated by a porous membrane. Unlike previously published co-culture systems, we have combined enteroids grown from *C57BL6j* mice with syngeneic bone marrow-derived macrophages to preclude potential allo-reactions between immune cells and epithelium. Transformation of intestinal crypts into proliferative enteroids was achieved by cultivation in Wnt3a-Noggin-R-Spondin-conditioned medium supplemented with ROCK inhibitor Y-27632. The differentiated phenotype was promoted by the use of the Wnt3-deprived EGF-Noggin-R-Spondin medium. The resulting co-culture of primary cells can be employed as a basic model to better understand the reciprocal relationship between intestinal epithelium and macrophages. It can be used for in vitro modelling of mucosal inflammation, mimicked by stimulation of macrophages either while being in co-culture or before being introduced into co-culture, to simulate enterogenic sepsis or systemic conditions affecting the intestinal tract.

## 1. Introduction

The intestinal epithelial barrier’s main function includes maintaining mucosal homeostasis, which is accomplished by a fine-tuned communication network composed of the epithelium, adaptive and innate immune cells, and luminal microbiota. Any perturbations within this microenvironment can compromise the epithelial border, followed by dysbiotic microbiota changes, bacterial translocation, and either short-lived flares or ongoing inflammatory activity within the intestinal wall [1]. Among innate immune cells residing in the subepithelial mucosa, tissue macrophages constitute one of the most abundant cell populations. Having differentiated from migratory bone marrow-derived monocytes, tissue macrophages are important drivers of acute and chronic inflammation through the release of pro-inflammatory cytokines, chemokines, and interferons [2,3,4]. For cell culture experiments involving primary cells, bone marrow monocytes provide a copious source to obtain macrophages through M-CSF-driven differentiation but require harvesting from a live organism and cannot be propagated. Under certain circumstances, they may be replaced by immortalized macrophage cell lines, including RAW 264.7 and NR-9456, which functionally and morphologically resemble primary macrophages but exhibit physiological traits different from non-transformed cells. For this study, we selected bone marrow-derived macrophages as the source of macrophages for the closest possible match with in vivo reality.

Murine colitis models are still regarded as a foundation of studies assessing the impact of a genetic manipulation or pharmacological intervention on the intestinal barrier function in a complex organism. At the same time, the in vivo experimental approach excludes the possibility of disentangling the crosstalk between specific cell populations to define their individual contribution to the inflammatory environment. This drawback is addressed by co-cultures typically combining intestinal epithelium with an additional cell population which commonly involves, but is not restricted to, polymorphonuclear cells [5,6], macrophages [6,7,8], and lymphocytes [9,10].

Initially, intestinal epithelial lining was created from intestinal cancer cell lines [11,12] or primary intestinal cells [13,14], both of which come with important downsides. Cancer cell lines show a loss of the phenotypical characteristics of non-transformed cells, whereas primary intestinal epithelial cells are prone to early apoptosis in the absence of their neighbouring feeder cells [13]. These limitations were surmounted by organoid bioengineering technology: three-dimensional miniature guts originating from adult stem cells (hereafter termed enteroids) recapitulate phenotypical and metabolic features of the adult intestinal epithelium and are fitting to study physiological and pathological processes ex vivo. Successful cultivation and propagation of enteroids rely on essential factors, including Noggin and R-Spondin, to stimulate intestinal stem cell growth and promote survival. These niche factors can be supplied either as recombinant growth factors [15] or growth factor-conditioned medium [16,17]. Recombinant growth factors ensure high purity and experimental consistency, whereas growth factor-conditioned medium is cost-wise affordable but confers variability in growth factor concentrations.

While initial attempts of cultivating enteroids were focused on murine intestinal stem cells, enteroids can be successfully grown from a variety of mammalian species, including *Homo sapiens* sp. *sapiens* [18,19,20,21]. The utilization of human enteroids adds particular clinical relevance to animal studies data and provides an option for personalized medical applications such as individual disease modelling from patient-derived intestinal biopsies. However, due to the invasiveness of endoscopic or surgical tissue sampling, access to human biomaterials is generally limited. Therefore, we propose a murine enteroid-based platform incorporating macrophages to serve as a reproducible co-culture system. Either freshly prepared or cryo-preserved enteroids and BMDM can be used for co-culture purposes. Using cryo-preserved material offers the advantage of circumventing the need to coordinate time-consuming procedures of enteroid cultivation and macrophage differentiation preceding the actual experiment.

This protocol article intends to provide practical advice to ease the workflow by elaborating on technical details that are often neglected but are key to improving efficiency. In addition to pointing out a way to split the workflow by using cryo-preserved cellular material for co-culture, it provides insight into culture and handling techniques necessary to ensure optimized post-freezing recovery rates of macrophages and enteroids.

Adhering to the steps outlined in this protocol will allow the construction of an enteroid–BMDM co-culture system, which can be used to examine reciprocal interactions between the intestinal epithelium and the innate immune cell system.

## 2. Materials

### 2.1. Animals and Cell Lines

- *C57BL/6j* mice were bred and housed under specific pathogen conditions at the University of Ulm’s animal research facility. Breeding pairs were initially obtained from the Jackson Laboratory. Five mice were housed per SPF cage at 21–24 °C room temperature, air humidity 42–44%, light/dark cycle of 14:10 h.
- L-WNR cells, stably expressing Wnt3a, Noggin, and R-Spondin 3, #S0011002, AddexBio, San Diego, Ca, USA.

### 2.2. Reagents and Chemicals

#### 2.2.1. General Requirements

- Fetal bovine serum (FBS), heat-inactivated, # F7524, Sigma-Aldrich, Taufkirchen, Germany.
- Dulbecco’s Phosphate Buffered Saline (DPBS), ‘# 14190-094, Gibco, Paisley, UK.
- Penicillin/Streptomycin, 10,000 units/mL/10 mg/mL, # 15140-122, Gibco, Paisley, UK.
- Dimethyl Sulfoxide (DMSO), # A3672, PanReac AppliChem, Darmstadt, Germany.
- Ethylenediaminetetraacetic acid disodium (EDTA), # E7889-100ML, Sigma-Aldrich, Darmstadt, Germany.
- Trypsin-EDTA (0.5%), # 15400054, Gibco, Paisley, UK.

#### 2.2.2. Isolation and Differentiation of Bone Marrow-Derived Macrophages

- Dulbecco’s Modified Eagle Medium (DMEM), supplemented with D-Glucose, L-Glutamine, Pyruvate, # 41966-029, Gibco, Paisley, UK.
- Recombinant mouse Macrophage Colony Stimulating Factor (M-CSF), # 315-02, PeproTech (part of Thermo Fisher, Weil am Rhein, Germany) (lot no. 0518245 A0220).
  o Reconstitute lyophilized recombinant M-CSF with sterile distilled H_2_0 (e.g., Ampuwa Plastipur) to obtain a stock solution of 100 µg/mL, aliquot at appropriate amounts (e.g., 10 µL), and store at −20 °C.
- Ethanol, # 32205, Sigma-Aldrich, Darmstadt, Germany.
- NH_4_Cl, # K298.2, Roth, Karlsruhe, Germany.
- Trizma-Base (Tris-Base), # 93362, Sigma-Aldrich, Darmstadt, Germany.
- Ampuwa Plastipur (sterile distilled H_2_O), # B230673, Fresenius Kabi, Bad Homburg, Germany.
- Lipopolysaccharide from Escherichia coli O55:B5, # L6529, Sigma-Aldrich, Darmstadt, Germany.
- RNeasy Mini Kit, # 74106, Qiagen, Hamburg, Germany.
- Primers: Mm_Tnf_1_SG QuantiTect Primer Assay, # QT00104006; Primer Assay for Mouse GAPDH primer, # PPM02946E-200, Hamburg, Qiagen, Germany.

### 2.3. Enteroid Culture

- L-WNR-conditioned medium. Prepared according to [16]). For steps of production please refer to “Media and Solutions Formulations”.
- Antibiotic-Antimycotic 100X, # 15240096, Gibco, Paisley, UK.
- Advanced DMEM/F12 (1x), # 12634-010, Gibco, Paisley, UK.
- GlutaMAX (100X), # 35050061, Gibco, Paisley, UK.
- B-27 Supplement (50X), # 12587010, Gibco, Paisley, UK (lot. no. 2584583).
- N-2 Supplement (100X), # 17502048, Gibco, Paisley, UK (lot. no. 2584679).
- Murine Epidermal Growth Factor (EGF), # 315-09, PeproTech (part of Thermo Fisher, Weil am Rhein, Germany) (lot. no. 0519179 L1219).
- R-Spondin 1, # 3474-RS-050, R&D Systems, Mineapolis, MN, USA (lot. no. OHO5420011).
- Murine Noggin, # 250-38, PeproTech (part of Thermo Fisher, Weil am Rhein, Germany) (lot. no. 0618407 J0318).
- Collagenase type I, # 17100-017, Invitrogen (part of Thermo Fisher, Weil am Rhein, Germany) (lot. no. 2445202).
- Gentamicin 50 mg/mL, # 15750060, Invitrogen (part of Thermo Fisher, Weil am Rhein, Germany).
- HEPES, 1 M, # 7365-45-9, Sigma-Aldrich, Darmstadt, Germany.
- Y-27632, # Y0503-1MG, Sigma-Aldrich, Darmstadt, Germany (lot. no. 0000310878).
  o Reconstitute lyophilized Y-27632 with sterile distilled H_2_O (e.g., Ampuwa Plastipur) to obtain a stock solution of 1 mM, aliquot at appropriate amounts (e.g., 100 µL), and store at −20 °C.
- Basement Membrane Matrix, # 354234, Corning, Bedford, MA, USA (lot. no. 3116001).
- CryoStor CS10, #07930, Stem Cell Technologies, Cologne, Germany.

### 2.4. Equipment

- Water bath: Bender & Hobein GmbH, München, Germany (set to 37 °C).
- Cell culture incubator: Heracell 150, Thermo Scientific, Weil am Rhein, Germany (set to 37 °C 5% CO_2_).
- Biosafety cabinet (S1): Herasafe 2025 1.8, Thermo Scientific, Weil am Rhein, Germany.
- Refrigerated centrifuge: Multifuge 3 S-R, Heraeus, Thermo Scientific, Weil am Rhein, Germany.
- CO_2_ rodent euthanasia chamber: # 51370, Otto Environmental, Greenfield, WI, USA.
- Hemocytometer: Neubauer improved, depth 0.1 mm, Marienfeld, Lauda-Königshofen, Germany.
- Stainless steel blunt-end, strait scissors, ball scissors, tweezers, and gavaging needles: Fine Science Tools GmbH, Heidelberg, Germany.
- TC-coated dishes of 10 mm and 100 mm: #150288 and #150350, Nunclon Delta Surface, ThermoFisher Scientific, Weil am Rhein, Germany.
- Polystyrene conical tubes measuring 15 and 50 mL: Falcon 15 mL and 50 mL, #352099 and #352070, Corning, Bedford, MA, USA.
- Cell strainers measuring 70 µm and 100 µm: Falcon cell strainer 70 µm and 100 µm, #352350 and #352360, Corning, Bedford, MA, USA.
- Disposable safety scalpels, feather-shaped: # BA810SU, Braun, Tuttlingen, Germany.
- Freezing container: Mr. Frosty, ThermoFisher, Weil am Rhein, Germany.
- Mortar and pestle (sterile): # GZ-63100-56 (capacity 125 mL), Cole-Parmer, Cambridgeshire, UK.
- Non-TC-treated Petri dishes: 100 × 20 mm Style dish, # 430591, Corning, Bedford, MA, USA.
- Plunger of a 5 mL syringe (sterile): # 4606051V, B. Braun Medical AG, Switzerland.
- Twenty-four-well plates: CellStar, #662160, Greiner bio-one, Darmstadt, Germany.
- Permeable supports: Transwell pore size 0.4 µm, # 3470, Corning, Bedford, MA, USA.

### 2.5. Media and Solutions Formulations

- DPBS-0: Add Antimycotic-Antibiotic to DPBS to obtain a final 1% concentration. Can be stored at 4° C for up to 4 weeks.
- Coating Solution: Add FBS to DPBS to obtain a final concentration of 1%. NOTE: Must be prepared fresh and used on the same day.
- Macrophage Basic Culture Medium: Add penicillin/streptomycin and FBS to DMEM medium to obtain a final 1% and 10% concentration, respectively. Can be stored at 4 °C for up to 4 weeks.
- Macrophage Complete Culture Medium: Add recombinant M-CSF to Macrophage Basic Culture Medium to obtain a final concentration of 20 ng/mL. Can be stored at 4 °C for up to 1 week.
- Red Blood Cell (RBC) Lysis Buffer: Dissolve NH_4_Cl (final concentration 144 mM) and Tris-Base (final concentration 17 mM) in distilled H_2_O. Adjust the pH to 7.2. Can be stored at 4 °C for up to 4 weeks.
- Macrophages Freezing Medium: Add FBS and DMSO to DMEM to achieve a final concentration of 20% and 10%, respectively.
- Enteroid Proliferation Medium (adapted from Stappenbeck et al. [16])*:* Briefly, L-WNR cells are maintained under dual antibiotic selection with hygromycin B and geneticin until 100% confluence is reached. Then, media is replaced and the conditioned medium is harvested on 4 consecutive days. Eventually, all batches are pooled together and sterile-filtered through a syringe filtration unit (pore size 0.2 µm) before storing at −20 °C. For detailed steps and additional reagents required please refer to the original protocol [16].
- CRITICAL: It is strongly recommended that the L-WNR-conditioned medium be supplemented with Y-27632 (final concentration 10 µM) when performing critical steps such as starting cultivation, passing, and thawing of enteroids.
- Enteroid Basic Medium: Add penicillin/streptomycin and GlutaMAX to Advanced DMEM/F12 to obtain a final concentration of 1% per supplement.
- Enteroid Differentiation Medium (adapted from Sato et al. [15])*:* On the day of experiment, mix Enteroid Basic Medium with B27 (final concentration 1x), N2 (final concentration 1x), Noggin (final concentration 100 ng/mL), EGF (final concentration 20 ng/mL), and R-Spondin 1 (final concentration 500 ng/mL). For the purpose of co-culture with BMDM, the Enteroid Differentiation Medium must additionally be supplemented with M-CSF (final concentration 20 ng/mL).

## 3. Experimental Procedures

### 3.1. Phase I

The average time allotted for phase I (preparation and freezing of enteroids and BMDM) is ~5 and 7 days, respectively.

#### 3.1.1. Isolation and Differentiation of Bone Marrow-Derived Macrophages

1. Euthanize the mouse and place it onto the surgical pad in the supine position. Sterilize the skin of the abdomen and hindlimbs with skin antiseptic.
2. Make a longitudinal incision along the medial hind limb to expose the muscles. Dissect and remove the muscles surrounding the femur with sharp scissors. Extend skin incision and tissue preparation proximally towards the pelvic hip joint and distally towards the ankle. Separate the femur from the pelvic girdle by exarticulating the hip joint. Separate the distal portion of the hind limb from the femur with a twisting–tugging movement to exarticulate the knee joint. Expose the ankle joint and perform the same twisting–tugging movement to separate the paw from the tibia.
3. Remove residual muscles and soft tissues from the femur and tibia by thoroughly rubbing the bone with a dry paper towel, avoiding inadvertently damaging the corticalis or breaking the bone.
4. CRITICAL: The articular surface should remain intact. Leaving the corticalis undamaged preserves a sterile environment inside the medullary cavity, decreasing the risk of contamination of the bone marrow cell culture.
5. Dip the femur and tibiae in a 15 mL conical tube filled with 70% ethanol. Transfer to the sterile culture safety cabinet and continue processing in a sterile environment. Remove the bones with flame-scarved tweezers and place them in a 10 cm TC-treated dish with ice-cold DPBS to remove residual ethanol.
6. Transfer bones with a tweezer into a sterile mortar, add 1–2 mL DPBS, and grind until the bone marrow cell extract’s colour turns red to white.
7. Mount a 70 µm cell strainer on top of a 50 mL polystyrene conical tube and pour bone marrow cell suspension, including bone fragments, onto the membrane.
8. Squeeze the bone marrow cells through the cell strainer with a sterile plunger of a 5 mL syringe, then rinse the strainer with 5 mL ice-cold DPBS.
9. Centrifuge bone marrow cells (400× *g*), 7 min, 4 °C and aspirate the supernatant.
10. Resuspend the bone marrow cell pellet with 1 mL RBC lysis buffer and incubate for 5 min at room temperature to lyse red blood cells. Stop the lysis reaction by adding 5 mL FBS.
11. Pelletize RBC-depleted bone marrow cells (400× *g*, 7 min, 4 °C) and remove the supernatant. Repeat steps 9 and 10 at least once to ensure near-complete removal of RBC.
12. Resuspend the cell pellet with 5 mL of Basic Macrophage Culture Medium and leave it on ice.
13. Count the bone marrow cells with a hemocytometer (optionally: check for cell viability with Trypan Blue staining) and adjust concentration for a final density of 5 × 10^6^ cells/mL in Macrophage Complete Culture Medium.
14. Seed bone marrow cells in non-TC-treated Petri dishes at a concentration of 5 × 10^6^ cells/mL Macrophage Complete Culture Medium to induce monocyte differentiation to macrophages.
15. CRITICAL: Tissue culture-treated dishes should be avoided for seeding bone marrow cells, as macrophages tend to adhere firmly to coated surfaces and can only be detached by applying mechanical forces, e.g., scraping them off with a rubber policeman.
16. Replace the Macrophage Complete Culture Medium every other day. Unlike other leukocyte fractions, monocytes remain attached to the dish while transforming into macrophages.
17. BMDM differentiation is expected to be completed by day 7. To release the BMDM, add 5–10 mL of DPBS supplemented with 10 mM EDTA to the dish and incubate for 10 min in the biosafety cabinet. Swirl the dish gently every 2–3 min to promote BMDM detachment.
18. Rinse the dish with a 10 mL pipet several times to release residual BMDM. As BMDM are accumulating, the EDTA/DPBS solution should adopt a turbid appearance. Collect the suspension in a 15 mL conical tube.
19. Centrifuge the BMDM suspension (400× *g*, 5 min, 4 °C). Resuspend the pellet in 250–500 µL Macrophage Complete Culture medium, dispense in aliquots, and freeze, or immediately use for co-culture experiments.

#### 3.1.2. Freezing of Mouse BMDM

1. Count the BMDM using a hemocytometer and centrifuge (400× *g*, 5 min, 4 °C).
2. Gently resuspend the cell pellet in freezing media to obtain a concentration of 5 × 10^6^ cells/mL.
3. Dispense 1 mL of BMDM suspension per cryovial and place it in a freezing container for gradual freezing. After 24 h, transfer to a liquid nitrogen tank for long-term storage.
4. Centrifuge (400× *g*, 5 min, room temperature) and remove the supernatant. Resuspend the BMDM pellet in the desired volume of Macrophage Complete Culture Medium or immediately use it to establish enteroid–BMDM co-culture.

#### 3.1.3. Thawing of Mouse BMDM

5. Thaw BMDM by immersing a cryovial in a 37 °C water bath until only an ice chunk is left, then immediately resuspend in a 15 mL conical tube filled with 10 mL pre-warmed Macrophage Basic Culture Medium to dilute residual DMSO.
6. Centrifuge (400× *g*, 5 min, room temperature) and remove the supernatant. Resuspend the BMDM pellet in the desired volume of Macrophage Complete Culture Medium or immediately use it for establishing enteroid–BMDM co-culture (continue with section “Establishing co-culture of enteroids and BMDM”).

#### 3.1.4. Cultivation of Mouse Enteroids

1. Euthanize the mouse and immobilize it in the supine position on a surgical pad. Sterilize the abdomen and hindlimbs with skin antiseptic.
2. Make a longitudinal incision with pointed scissors to open the peritoneal cavity. Remove the pancreas and spleen. Separate the duodenum from the stomach at the gastric-intestinal junction. Stretch the small intestine to its full length and separate at the transition to the cecum. Remove as much mesenterial tissue from the intestine as possible.
3. Place the isolated small intestine in a 10 cm dish with an ice-cold DPBS-0 and flush it using a 20 mL syringe fitted with a stainless-steel gavaging needle to remove large fecal matter.
4. Slice the intestine longitudinally using a ball scissor and cut it into 3–4 pieces.
5. Transfer the intestinal fragments to a new 10 cm dish filled with ice-cold DPBS-0 and wash it vigorously. Repeat the washing step.
6. Transfer the intestinal fragments to a new 10 cm dish filled with ice-cold DPBS-0 and incubate for 15 min at 4 °C with gentle agitation on a rotating shaker (set speed to 100 rpm). Repeat this step once more.
7. CRITICAL: From that point, work must be carried out under laminar flow to maintain sterile conditions.
8. Place the intestinal fragments in an empty 10 cm dish and mince them finely into a mash using two scalpels.
9. CRITICAL: From now on, all dishes and pipettes that come in contact with the minced tissues must be pre-coated with Coating Solution to avoid sticking and tissue loss.
10. Transfer the mash to a pre-coated 50 mL conical tube filled with 20 mL DPBS-0 and invert 20 times. Allow the tissue pieces to settle to the bottom of the tube before the supernatant is aspirated. Then, replenish with 10 mL cold DPBS-0 and repeat Step 10 at least 5 times until the supernatant has become clear.
11. Aspirate supernatant and resuspend the pellet in 30 mL of 2 mM EDTA/DPBS-0. Incubate at 4 °C for 30 min on a rotating shaker.
12. Let the pellet settle at the bottom of the tube and remove the supernatant.
13. Add 10 mL DPBS-0 and, using a Pipet boy, forcefully aspirate the tissue fragments at maximum pipetting speed and release slowly by gravity dispense (repeat this procedure 10 times). Let the pellet settle and discard the supernatant (fraction I: contains villi).
14. Repeat step 11 and discard the supernatant (fraction II: contains both villi and crypts).
15. Repeat step 11 to obtain fraction III enriched in crypts. Immediately filter the supernatant through a 100 µm cell pre-coated strainer mounted on a 50 mL pre-coated conical tube. Keep the flow-through.
16. Add another 10 mL DPBS-0 to the remaining tissue fragments and shake vigorously to release residual crypts. Pour the entire suspension through the same 100 µm cell strainer as indicated in step 13.
17. Centrifuge the crypt suspension (400× *g*, 5 min, 4 °C) and remove the supernatant.
18. Carefully resuspend the crypt pellet in 10 mL Advanced DMEM/F12 medium and filter through a 70 µm cell strainer.
19. Centrifuge the crypt-enriched flow-through (400× *g*, 5 min, 4 °C), remove the supernatant, and place the tube on ice.
20. Resuspend the crypt pellet in an appropriate amount of Matrigel aiming at 15–20 µL of Matrigel per well, and mix gently.
21. Place a droplet of 15–20 µL crypt–Matrigel suspension in the centre of a well of a 24-well plate and let polymerize in the incubator (20 min, 37 °C, 5% CO_2_).
22. Overlay each crypt–Matrigel dome with either 500 µL Enteroid Proliferation Medium or Enteroid Differentiation Medium supplemented with Y-27632 and cultivate at 37 °C, 5% CO_2_.
23. Change medium after 2 days.
24. NOTE: On day 5, intestinal crypts are expected to have developed into cyst-like (Enteroid Proliferation Medium) or crypt-like structures (Enteroid Differentiation Medium). At this stage, both types of enteroids require routine passaging (continue with section “Passaging of mouse enteroids”). Additionally, enteroids with a cyst-like phenotype can be cryopreserved for future use (continue with section “Freezing of enteroids”).

#### 3.1.5. Passaging of Mouse Enteroids

1. Aspirate the medium and add 500 µL DPBS-0 to rinse the wells.
2. Add 200 µL Trypsin-EDTA per well and keep the plate in the incubator at 37 °C for 3 min.
3. Add 500 µL washing medium and lift off the enteroid–Matrigel domes by scratching horizontally and vertically with the tip of a 10 mL pipet.
4. Dissociate enteroids by vigorous pipetting 5 times up and down with a 1000-µL pipet tip.
5. Collect enteroid suspension from all wells with a 10 mL pipet and transfer it to a 50 mL conical tube.
6. Attach a 1000 µL pipet tip to a 10 mL tip and pipet up and down 5 times to break down enteroids into small fragments and single cells.
7. Centrifuge the resulting suspension (400× *g*, 5 min, 4 °C).
8. Aspirate the supernatant and resuspend the pellet with 5 mL washing medium. Transfer the suspension to the 15 mL conical tube.
9. Centrifuge the enteroid suspension (400× *g*, 5 min, 4 °C).
10. Aspirate the supernatant and place the enteroid pellet on the ice.
11. Resuspend the enteroid pellet in an appropriate volume of Matrigel aiming at 15–20 µL of Matrigel per well, and mix gently.
12. Plate a droplet of 15–20 µL of crypt–Matrigel suspension in the centre of a well of a 24-well plate and let polymerize in the incubator (20 min, 37 °C, 5% CO_2_).
13. Overlay each crypt–Matrigel dome with 500 µL Enteroid Proliferation Medium or Enteroid Differentiation Medium supplemented with Y-27632 and cultivate at 37 °C, 5 CO_2_.
14. Change medium after 2 days or freeze enteroids (continue with section “Freezing of enteroids”).

#### 3.1.6. Freezing of Mouse Enteroids

1. Depending on the size of the enteroid pellet, add an appropriate volume of CryoStor freezing medium and mix thoroughly.
2. Dispense 1 mL of the enteroid suspension per cryovial and place it in a freezing container for gradual cooling down to −80 °C overnight. Then, transfer to a liquid nitrogen tank for long-term cryo-storage.

#### 3.1.7. Thawing of Mouse Enteroids

1. Thaw enteroids by immersing a cryovial in a 37 °C water bath until only a few ice chunks are left, then immediately resuspend in 10 mL pre-warmed Advanced DMEM/F12.
2. Centrifuge (400× *g*, 5 min, room temperature) and remove supernatant
3. Resuspend the enteroid pellet in the desired amount of Matrigel (continue with the section “Establishing co-culture of enteroids and BMDM”).

### 3.2. Phase II

Establishing co-culture of mouse enteroids and BMDM

1. Follow the instructions in the section “Thawing of mouse enteroids”.
2. Resuspend the enteroid pellet in an appropriate volume of Matrigel aiming at 15–20 µL of Matrigel per well, and mix gently.
3. Plate a droplet of 15–20 µL of crypt–Matrigel suspension in the centre of a well of a 24-well plate and let polymerize in the incubator (20 min, 37 °C, 5% CO_2_).
4. Cover each crypt–Matrigel dome with 500 µL Enteroid Proliferation Medium supplemented with 10 µM Y-27632 and incubate for 3 h at 37 °C, 5 CO_2_.
5. CRITICAL Immediately after thawing, enteroids tend to be fragile and thus should first be exposed to Enteroid Proliferation Medium for a minimum of 3 h to speed up their recovery from liquid nitrogen. Subsequent differentiation/crypt budding of the enteroids is promoted by replacing Enteroid Proliferation Medium with Enteroid Differentiation Medium devoid of Wnt3a.
6. Meanwhile, defrost BMDM following the instruction in the section “Thawing of BMDM”, then immediately place on ice.
7. Determine the concentration of viable BMDM by counting Trypan blue-negative cells using a Neubauer hemocytometer. Suitable BMDM numbers for co-culture are ranging between 10^5^–10^6^ cells per Matrigel dome.
8. Centrifuge the BMDM suspension containing the required absolute number of cells (400× *g*, 5 min, 4 °C) and aspirate the supernatant.
9. Resuspend the BMDM pellet with the appropriate volume of Enteroid Differentiation Medium supplemented with M-CSF (20 ng/mL) and mix by gently pipetting up and down.
10. NOTE: For a Transwell insert compatible with a 24-well plate, calculate a volume of 200 µL.
11. Dispense 200 µL of BMDM suspension to each Transwell insert and position above the already solidified Matrigel dome containing the enteroids to enable close contact between the cell populations. Preferably cultivate at 37 °C, 5 CO_2_ overnight before co-culture experiments can be carried out.

## 4. Results and Discussion

Gut immunology is a field of growing interest as the intestinal barrier may either be affected by local inflammatory conditions or involved in a systemic disorder such as sepsis or other critical conditions. Gaining a deeper understanding of the crosstalk between the intestinal epithelium and immune cells is crucial in identifying molecular targets that could be exploited to attenuate the inflammatory response or promote epithelial regeneration following acute injury. Intestinal co-cultures represent a broadly accepted model suitable for studying the interaction between the epithelium and specific immune cells, undisturbed by the multicellular mucosal environment. To simplify and accelerate co-culture-based experiments, we established a robust co-culture platform combining murine enteroids and BMDM in separate niches.

**Figure 1:**
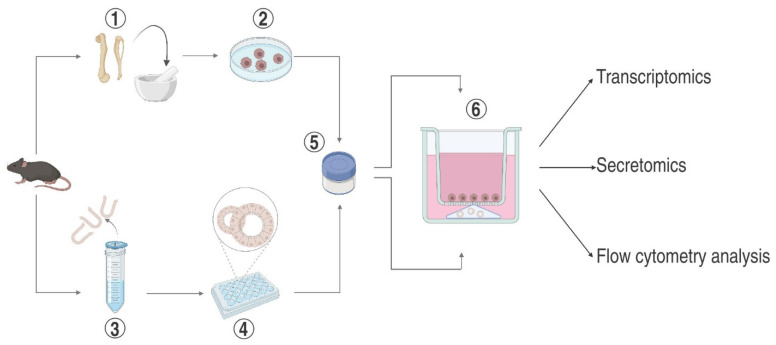
Outline of key steps in enteroid cultivation and BMDM differentiation and potential fields of application: (**1**) Surgical dissection of tibia and femur, (**2**) extraction of bone marrow cells and mCSF-dependent selection and differentiation of monocytes into macrophages, (**3**) harvesting of the small intestine and release of crypts, (**4**) cultivation of embedded crypts with Enteroid Proliferation Medium, (**5**) liquid nitrogen storage, and (**6**) assembly of BMDM and enteroids into co-culture.

A few protocols addressing non-cancerous intestinal co-culture systems are available, most of which are based on the compartmentalization of enteroid-derived epithelium and different types of immune cells in a semi-permeable Transwell system. Our protocol is suited for experiments in which a preserved 3D architecture of the enteroids is desirable. Conventionally, enteroids are dissociated into single cells and seeded on the Transwell insert membrane, resulting in monolayer formation and loss of structural dimensionality. Our approach can be used to probe the impact of quiescent or activated BMDM on enteroids; both populations remain strictly segregated, which facilitates the harvesting and analysis of single cell types. Due to the composite design, our system gains versatility as co-cultivation can easily be terminated or interrupted by removing the BMDM-containing Transwell insert and continuing enteroid culture alone. Media from either compartment may be sampled separately to determine gradients of soluble mediators secreted by BMDM or epithelial cells (Figure 1).

**Figure 2:**
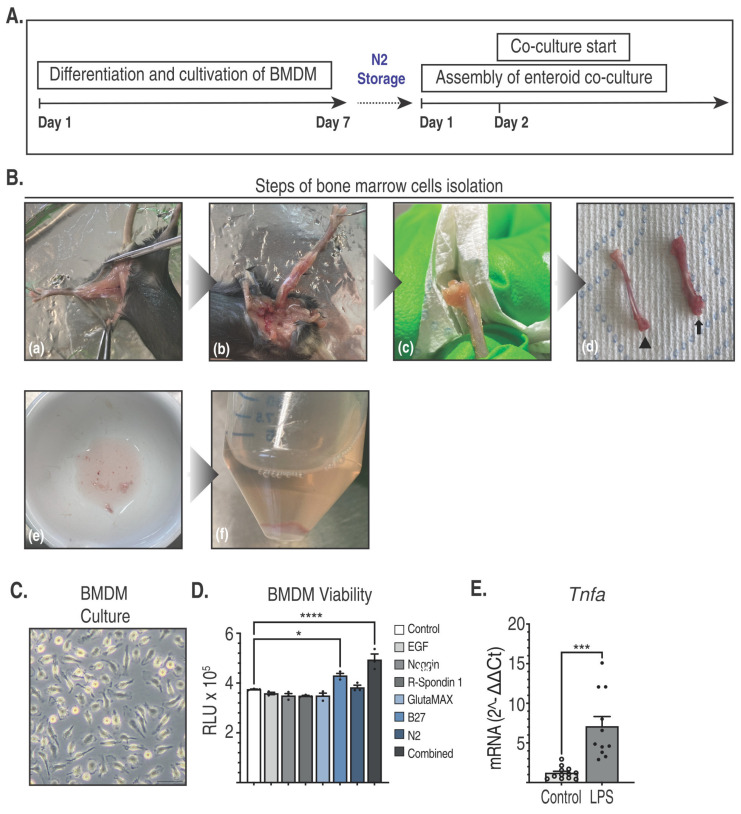
(**A**) Timeline of BMDM cultivation and incorporation into co-culture. (**B**) Illustration of sequential steps of bone marrow cell isolation: (**a**) Surgical exposure of the hindlimb muscles. (**b**) Dissection of the biceps femoris, semitendinosus, gracilis, and adductor muscles. (**c**) Removal of residual soft tissues from the bone by rubbing with a paper towel. Articulating surfaces of bones should remain intact to avoid medullary contamination. (**d**) Denuded femur (black arrow) and tibia (black arrowhead). (**e**) Crushing of bones to release bone marrow cells. (**f**) Sedimented bone marrow cells depleted from RBC post-lysis. Representative brightfield image of BMDM growing on a TC-coated dish 24 h after thawing. Scale bar 50 µm (**C**). Viability of BMDM treated with single and combined agents included in Enteroid Differentiation Medium at the indicated concentrations (n = 3 independent experiments). Control: Enteroid Basic Medium, EGF: 20 ng/mL, Noggin: 100 ng/mL, R-Spondin 1:500 ng/mL, GlutaMAX: 2.5×, N2: 1×, B27 1×, Combined: Enteroid Differentiation Medium. Viability was determined by measuring intracellular ATP (CellTiter Glo 2.0, Promega, Walldorf, Germany. Luminescence readings were expressed as relative light units (RLU). Statistical analysis was performed using one-way ANOVA followed by Dunnett’s multiple comparisons test (**D**). Immune responsiveness of BMDM. Thawed BMDM were seeded at a density of 10^5^ cells per well in a 12-multiwell plate. Transcriptional expression of TNFα was determined following 24 h of in vitro stimulation with 500 ng/mL LPS versus DPBS control. Statistical analysis was performed using an unpaired *t*-test (**E**), * *p* < 0.05, *** *p* < 0.001, **** *p* < 0.0001.

Crucially, we propose a co-culture platform which instead of using freshly cultured enteroids can be composed of previously cryopreserved enteroids. Once assembled with BMDM, it is ready for use within less than 24 h. Instead of a two-stage procedure which includes instant freezing of bone marrow cells and resuming differentiation into macrophages at a later point, we opted for cryopreservation of mature BMDM. The selective survival and differentiation of monocytes into macrophages were induced by adding CSF to freshly isolated bone marrow cells which were maintained in culture for 7 days (Figure 2A,B). For seeding the bone marrow cell mixture, we chose non-tissue-coated Petri dishes to facilitate the later release of intact BMDM from the surface.

The viability of BMDM was not impaired by long-term cryostorage which could be maintained for at least three years. By 24 h after thawing and plating, nearly all BMDM have firmly attached to the dish surface, adopting a typically spindle-like elongated shape (Figure 2C). Before introducing BMDM into co-culture, in vitro tolerance towards the ingredients of the enteroid medium was tested by exposing BMDM to EGF, R-Spondin 1, Noggin, Glutamax, N2, B27, and Enteroid Differentiation Medium. Enteroid Basic Medium served as a control condition. The viability of BMDM was not substantially impaired by cultivation in the presence of Enteroid Differentiation Medium ingredients. Conversely, BMDM viability was significantly increased in response to B27 and Enteroid Differentiation Medium, indicating that BMDM can thrive in the presence of enteroid culture medium (Figure 2D). BMDM were responsive to in vitro stimulation with lipopolysaccharide, showing significant upregulation of TNFα expression (Figure 2E).

**Figure 3:**
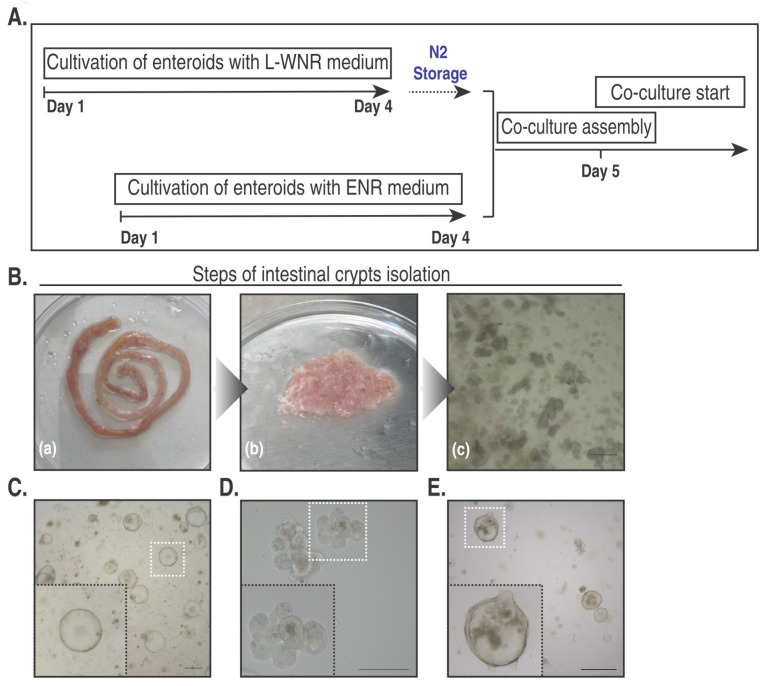
(**A**) Timeline of enteroid cultivation and incorporation into co-culture. (**B**) Illustration of sequential steps of intestinal crypt isolation: (**a**) Small intestine after forceful flushing with DPBS to remove large fecal matter. (**b**) Finely minced intestinal tissue prior to incubation with EDTA chelating buffer. (**c**) Single intestinal crypts after sequential filtration through a 100 and 70 µm cell strainer, respectively. Scale bar 100 µm. (**C**) Representative brightfield image of proliferative enteroids with a cystic morphology at 96 h of cultivation in Wnt3a-containing Enteroid Proliferation Medium). Scale bar 500 µm. (**D**) Representative brightfield image of differentiated hyper-branching enteroids at 96 h of cultivation in Wnt3-deprived Enteroid Differentiation Medium. Scale bar 100 µm. (**E**) Representative brightfield image showing enteroids with a largely preserved cystic shape interspersed with nascent buds 24 h after thawing, cultivated in Wnt3a-deprived Enteroid Differentiation Medium. Scale bar 500 µm. Insets are enlarged images of the respective dashed areas.

Liberation of small intestinal crypts from the underlying lamina propria was most efficiently achieved by EDTA, as proposed by Sato et al. [15,22]. The crypt isolation procedure, as outlined in the protocol (Figure 3A,B), was adapted from Vignjevic et al. [22]. After seeding, intestinal crypts require ~96 h of culture to develop into enteroids, which can be further propagated or cryostored. When cultivated in the Enteroid Proliferation Medium, enteroids remain highly proliferative, appearing as large thin-walled spheres (Figure 3C). As opposed to differentiated enteroids (Figure 3D), such enteroids are better adapted to freezing owing to a Wnt3a-mediated expansion of the stem cell niche, facilitating a speedy recovery after thawing. To avoid disruption and early apoptosis of enteroids, overall handling time in cell culture should not exceed 1 h. Enteroids are transiently exposed to Enteroid Proliferation Medium for at least 3 h to ease the transition between thawing and cultivation; subsequent cultivation is continued with Enteroid Differentiation Medium. Upon withdrawal of Wnt3a, enteroids start forming small protrusions, which precede the later budding of crypts (Figure 3E).

However, as opposed to the 2D co-culture format, in vitro permeability studies such as transepithelial electric resistance measurements cannot be easily realized in our co-culture but require an indirect approach by adding FITC-conjugated dextran molecules at a defined concentration to the media and recording changes in fluorescence intensity inside and outside the enteroid lumen with time-lapse imaging. Although enteroids and BMDM are brought in proximity to one another, our co-culture setup hinders immediate cell–cell contact, making it an impracticable environment to investigate ligand-dependent interactions.

## 5. Conclusions

With this protocol, we intend to facilitate the implementation of intestinal co-culture projects. Owing to the system’s composite design, macrophages can be selectively challenged before being brought together with the intact intestinal epithelium, simulating the influx of activated macrophages into the otherwise healthy intestinal mucosa. Therefore, our co-culture system can be used to function as a model of acute intestinal injury which may be a collateral event in systemic critical conditions such as polytrauma or extensive burns. Likewise, it is suitable to model enterogenic sepsis by stimulating macrophages with lipopolysaccharide, a characteristic pathogen-associated molecular pattern of Gram-negative bacteria. Another strength of this co-culture system includes the use of enteroids and macrophages with a syngeneic background to preclude potential allo-reactions between immune cells and epithelium.

By utilizing cryo-preserved enteroids and BMDM, we introduce a pause point between the generation and processing of cellular elements which can be extended at the researcher’s discretion. Lowering the hurdles for otherwise time-consuming contiguous procedures such as co-culture experiments will hopefully encourage further studies addressing the interface of the intestinal epithelium and the innate immune cell system.

## Abbreviations

FBS	Fetal Bovine Serum
BMDM	Bone Marrow-Derived Macrophages
DPBS	Dulbecco’s Phosphate Buffered Saline
DMEM	Dulbecco’s Modified Eagle Medium
DMSO	Dimethyl Sulfoxide
EDTA	Ethylenediaminetetraacetic Acid Disodium
EGF	Epidermal Growth Factor
M-CSF	Mouse Macrophage Colony Stimulating Factor
RBC	Red Blood Cell
ROCK inhibitor	Rho-Associated Protein Kinase Inhibitor
SPF	Specific Pathogen Free
